# Joint extraction of entity and relation based on fine-tuning BERT for long biomedical literatures

**DOI:** 10.1093/bioadv/vbae194

**Published:** 2024-12-05

**Authors:** Ting Gao, Xue Zhai, Chuan Yang, Linlin Lv, Han Wang

**Affiliations:** School of Information Science and Technology, Northeast Normal University, Changchun 130117, China; School of Information Science and Technology, Northeast Normal University, Changchun 130117, China; School of Information Science and Technology, Northeast Normal University, Changchun 130117, China; Institute of Computational Biology, Northeast Normal University, Changchun 130117, China; School of Information Science and Technology, Northeast Normal University, Changchun 130117, China; School of Information Science and Technology, Northeast Normal University, Changchun 130117, China; Institute of Computational Biology, Northeast Normal University, Changchun 130117, China

## Abstract

**Motivation:**

Joint extraction of entity and relation is an important research direction in Information Extraction. The number of scientific and technological biomedical literature is rapidly increasing, so automatically extracting entities and their relations from these literatures are key tasks to promote the progress of biomedical research.

**Results:**

The joint extraction of entity and relation model achieves both intra-sentence extraction and cross-sentence extraction, alleviating the problem of long-distance information dependence in long literature. Joint extraction of entity and relation model incorporates a variety of advanced deep learning techniques in this paper: (i) a fine-tuning BERT text classification pre-training model, (ii) Graph Convolutional Network learning method, (iii) Robust Learning Against Textual Label Noise with Self-Mixup Training, (iv) Local regularization Conditional Random Fields. The model implements the following functions: identifying entities from complex biomedical literature effectively, extracting triples within and across sentences, reducing the effect of noisy data during training, and improving the robustness and accuracy of the model. The experiment results prove that the model performs well on the self-built BM_GBD dataset and public datasets, enabling precise large language model enhanced knowledge graph construction for biomedical tasks.

**Availability and implementation:**

The model and partial code are available on GitHub at https://github.com/zhaix922/Joint-extraction-of-entity-and-relation.

## 1 Introduction

Biomedical research is booming, automatically extracting entities and their relations from massive biomedical literatures has attracted much research attention due to its important applications on knowledge acquisition and construction. But extracting useful information from the huge library of scientific documents is laborious and time-consuming. And the information extraction and processing are very strict and accurate, which effectively prevent the pollution of the knowledge base, especially in the field of biomedicine.

In the 20th century, deep learning techniques are powerful for processing and understanding complex data: images, audio, or text. Large language model (LLM), such as generative pre-trained transformer (GPT) and bidirectional encoder representations from transformers (BERT), is efficient in text understanding, text generation, and text classification. These models learn rich language and knowledge representations through pre-training on large-scale text corpora, and tackle complex tasks of literature analysis in a variety of fields, especially in biomedical fields. However, LLM as black box model, is often unable to capture factual knowledge, and hallucination problems occur in the process of prediction and reasoning (Wen *et al.* 2023).

Knowledge graph (KG) (Zhang *et al.* 2021), a structured knowledge representations, has been widely used in many fields such as recommendation system, question answering system and web search. It represents various relations between entities in the form of graph, providing a way to understand the real world. However, there are limitations in terms of scale and timeliness, because the construction of KG often relies on manual annotation and expert knowledge. The existing KG have shortcomings in processing incomplete KG and constructing KG by processing text. In addition, the traditional KG often encounters incomplete knowledge coverage and lagging update.

Therefore, researchers try to integrate the LLM and KG to overcome various difficulties and limitations(Wen *et al.* 2023). LLM is applied to automatically extract entities and relations from text and add triples to the KG. LLM also generates text to enrich KG entity information and support complex question answering and reasoning by understanding KG’s structured data. These integrations improve the automation and expansion of KG, as well as its reasoning ability and information richness in practical applications.

A joint extraction of entity and relation model for biomedicine is proposed in this work. The model automatically extracts entities and their relations from scientific and technological literatures by deep learning and LLM-enhanced KG construction, and integrate the extracted information into a structured knowledge base. This database is useful for downstream tasks to build KGs in specific biomedical fields. The model not only helps biomedical researchers to obtain the core knowledge of the target literature more quickly and easily, but also plays an important role in the maintenance and construction of KG. The core tasks of the model include:

1. Text classification: Using fine-tuning BERT model for pre-training on large-scale literature corpus, the model implements the task of named entity recognition, classify literature in various fields, and analyze complex tasks of biomedicine.
2. Joint extraction of entity and relation: the model accurately identifies entities in the literature by using deep learning technology and graph convolutional network (GCN), and further analyze and extract the relations between entities based on the identification of entities.
3. Knowledge integration: The entities and relations obtained by joint extraction are integrated into the information in the form of triples, which is a foundation of the KG construction and enriches the content of the KG for subsequent downstream tasks.

Triplet datasets of biomedicine are constructed, and the model is verified on multiple public datasets in this work. The experiment results show that the model not only has excellent performance on self-built BM_GBD dataset, but also get good results on general datasets. The result proves that deep learning technology has great potentiality in biomedical literature analysis and KG construction in the context of current LLM.

This research not only improves the efficiency and accuracy of biomedical literature analysis by applying LLM-enhanced KG and deep learning techniques, but also opens up new avenues for automated construction and expansion of KG. This represents a broad prospect of cross-application of deep learning technology and biomedicine in the process of integration and promotion of LLM.

## 2 Methods

### 2.1 Development status of fusion of LLM and KG

The existing KG can be divided into four groups based on the information: (i) Wikipedia KG, (ii) common KG, (iii) domain-specific KG, (iv) multimodal KG. The domain-specific KG is represented by knowledge in a particular domain, which smaller in scale compared with others, but more accurate and reliable. UMLS (Bodenreider 2004), a domain-specific KG contains biomedical concepts and their relationships.

Pre-trained LLM shows great potential in various tasks in the field of NLP (Yang *et al.* 2023). LLM is divided into three groups: (i) encoder-only LLM, (ii) encoder-decoder LLM, (iii) decoder only LLM. Encoder-only LLM only uses encoders to encode sentences and understand the relationships between words. BERT (Devlin *et al.* 2018) belongs to encoder-only LLM, which is most effective for tasks that require understanding entire sentences, such as text classification (Yao *et al.* 2019) and named entity recognition (Santos and Guimaraes 2015).

**Figure 1.**
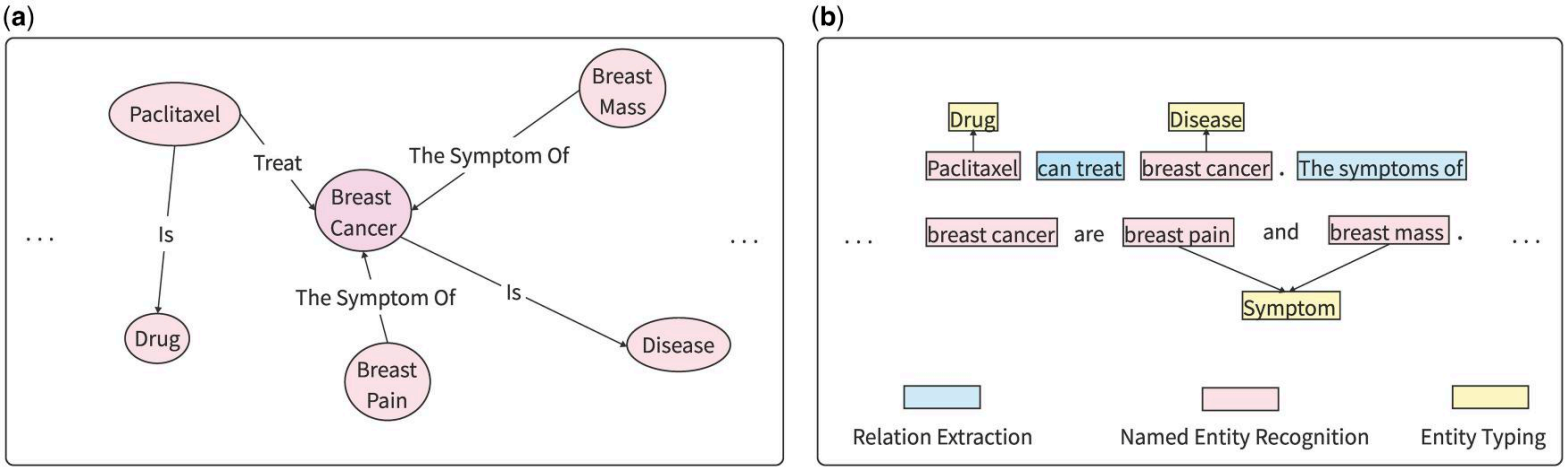
Comparison of the process of simple KG construction and LLM-enhanced KG construction. (a) KG. (b) LLM-enhanced KG construction.

The LLM-enhanced KG is adapted to various downstream tasks in recent research (Wen *et al.* 2023). Figure 1 shows the comparison of the process of KG construction and LLM-enhanced KG construction. Text is “Paclitaxel can treat breast cancer. The symptoms of breast cancer are pain and breast mass.” Figure 1a is a KG with breast cancer as the central entity, in which there are only entities and relations. Figure 1b is LLM-enhanced KG construction, which adds the classification of entities on the basis of entities and relations. Scientists try to use the text understanding and generation capabilities of LLM to automate the construction and expansion of KG. For example, entities and their relations are automatically extracted from scientific literature through LLM, and then the information is added to the KG in the form of triples. In addition, LLM enriched entity information by generating descriptive text, or supported complex question answering and reasoning tasks by understanding structured information in the KG.

### 2.2 Named entities recognition and relation extraction

In the late 1990s, a series of machine learning methods were applied on named entities recognition, and the named entities recognition system Kudo (Yamada and Matsumoto 2001) came out based on the supported vector machine (SVM) in 2001. However, machine learning only simplified the process of data classification, without simplifying feature extraction. Deep learning has been widely applied in entity extraction in recent decade. In 2011, Collobert *et al.* (2011) proposed a neural network architecture and learning algorithm based on Convolutional Neural Network (CNN) that was applied to various natural language processing tasks including part-of-speech tagging, chunking, named entity recognition, and semantic role labeling. Instead of exploiting artificial input features carefully optimized for each task, they learned internal representations on the basis of vast amounts of mostly unlabeled training data. But the problem of remote dependence between labels was not solved. In 2015, Santos and Guimaraes (2015) proposed a language-independent NER system that used automatically learned features only. In 2020, they evolved the char-CNN based on evolutionary deep learning techniques(Londt *et al.* 2020), which used word-level and character-level representations to perform sequential classification.

In 2015, Huang *et al.* (2015) proposed a variety of Long Short-Term Memory (LSTM) for sequence tagging. They attempted to apply a bidirectional LSTM conditional random field (denoted as BI-LSTM-CRF) on NLP benchmark sequence tagging datasets. In 2016, Chiu and Nichols (2016) presented a novel neural network architecture that automatically detected word and character-level features using a hybrid bidirectional LSTM and CNN. They also proposed a method of encoding partial lexicon matches in neural networks. In 2017, Rei (2017) presented a sequence labeling framework with a secondary training objective, learning to predict surrounding words for every word in the dataset. The entity extraction method was based on Transformer in 2017 (Vaswani *et al.* 2017). In 2021, the non-autoregressive set prediction method was applied on named entity recognition by Ye *et al.* (2021).

Most of the relationship extraction was obtained by statistical methods and machine learning, which all relied on artificial prior knowledge acquisition. Then the recurrent neural network (RNN), syntactic dependency tree, and word dependency matrix were applied for relation extraction. However, entity extraction and relation extraction were separate.

### 2.3 Joint extraction of entity and relation

In general, entity extraction is performed first and then relation extraction is performed. Errors in entity extraction are usually accumulated to the relationship extraction task, which lead to more cumulative errors in the downstream tasks. Therefore, the method of joint extraction appears. The purpose of joint extraction is to obtain the corresponding entities directly from the given text and extract the relations between the entities.

In 2016, Miwa and Bansal (2016) presented an end-to-end neural model to extract entities and relations. Their model captured both word sequence and dependency tree substructure information by stacking bidirectional tree-structured LSTM-RNNs on bidirectional sequential LSTM-RNNs. Since both methods were independent, they were not joint extraction in the true sense. In 2017, Li *et al.* (2017) replaced the original greedy-search decoding with beam-search and train the model with early-update techniques. Katiyar and Cardie (2017) presented a novel attention-based RNN for joint extraction of entity and relation. They did relate optimization work to reduce the error rate of entity extraction in the joint extraction process. In the same year, Zheng *et al.* (2017) proposed a joint extraction based on a novel tagging scheme to address the problem of inaccurate word label classification. In 2020, TPLinker (Wang *et al.* 2020) was introduced, which was a novel token pair linking mechanism and handshaking tagging scheme to jointly extract entities and relations in a single stage. But the model’s computational complexity was increased with longer sentences. In 2021, Zhong and Chen (2020) proposed a simple pipelined approach, which was built on two in dependent encoders and merely used the entity model to construct the input for the relation model. In 2022, Shang *et al.* proposed OneRel (Shang *et al.* 2022), which transformed entity and relation extraction into a fine-grained triplet classification task using a scoring-based approach and a Horns tagging strategy. But it struggled with processing highly complex datasets involving multiple nested entities, potentially limiting performance. In 2023, OD-RTE (Ning *et al.* 2023) combined object detection techniques with relational triple extraction using vertex-based encoding and a bidirectional diagonal decoding algorithm, but which is not suitable for entity extraction.

## 3 Results

**Figure 2.**
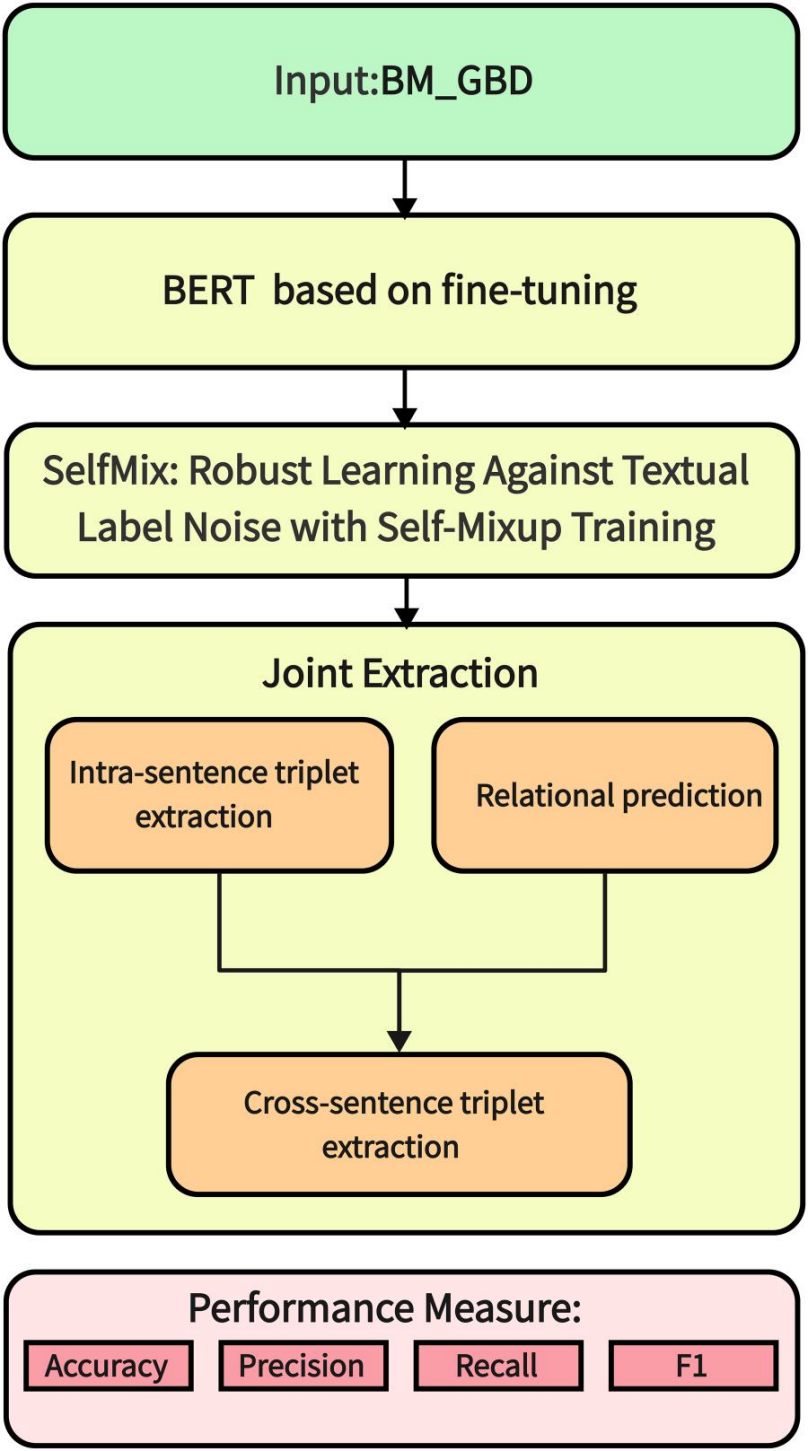
Flow of joint extraction.

Joint extraction of entity and relation system is designed in this study. Joint extraction follows three tasks: text classification, noise data processing and joint extraction. BERT (Devlin *et al.* 2018) model based on fine tuning is applied on dataset for named entity recognition and sentence pair relationship prediction. The SelfMix is used for noise data processing. The process of joint extraction implements intra-sentence triplet extraction and cross-sentence triplet extraction. Figure 2 describes the flow of the joint extraction system.

### 3.1 Sci BERT

BERT focuses on the global data processing medical or biological documents. Because the pre-training deep bidirectional models capture rich semantic information, which is essential for understanding specialized terms and complex concepts in the medical or biological field. The fine-tuning BERT models adapt to domain-specific semantic features, and exhibit higher accuracy in classification tasks. BERT is very suitable for specific datasets and tasks, so the model enhances the performance and efficiency of text classification and information extraction.

During the fine-tuning process, BERT undergoes additional training on domain specific datasets, which differs from initial pre-training. For the document classification in the biomedical science field, fine-tuning will adapt the model to the characteristics of a particular dataset. When dealing with biomedical science literature, it focuses on technical terms and complex sentence structures. Fine-tuning BERT model improves the model’s understanding of professional texts. Learning rate adjusted here is usually smaller than that in pre-training to avoid destroying the knowledge already learned by the model, which captures and understands the semantic features and context information of the target domain. During the fine-tuning process, all parameters of the model, from the embedding layer to the output layer, are updated according to the new dataset, and the model adapts to the new context. In text classification tasks, a specific classification tag header is typically added to the output layer of BERT, and the BERT model contains a fully connected layer that is optimized for a specific task. When entering text, special tags “[CLS]” and “[SEP]” are inserted to accommodate specific classification tasks, where the “[CLS]” tag is used to capture the overall meaning of the sentence, and the “[SEP]” tag is used to separate different sentences or paragraphs.

In the text classification of this study, we pay attention to the output vector of the “[CLS]” tag because it contains semantic information in the sentences or document. The maximum length is set to 512 in the experiment in order to maximize the effect of BERT. But the batch size is reduced appropriately in the experiment, because more resources are required during the training process. The hyperparameter and experiment results based on the fine-tuning BERT model (Sci BERT) are shown in Table 1.

**Table 1.** Hyperparameters based on fine-tuning BERT (Sci BERT).

Parameters	Value range
Batch Size	4 or 1
Dropout Parameters	0.2
BERT parameters are frozen	No
optimizer	RAdam
Learning rate	2e-5

### 3.2 SelfMix: robust learning against textual label noise with Self-Mixup training

When collecting and labeling data, annotation noise occurs in the dataset, which will adversely affect the fitting of the model. SelfMix presents a simple yet effective method to handle label noise in text classification tasks (Qiao *et al.* 2022). SelfMix uses the Gaussian Mixture Model to separate samples and leverages semi-supervised learning. The model not only achieves random sampling effect for negative samples, but also gets significant proportion of positive samples while retaining a large number of positive samples.

The core of SelfMix is to adapt noise in data through an innovative preprocessing method(Qiao *et al.* 2022). In this study, the BM_GBD dataset is independently obtained from the open source dataset and labeled on a semi-supervised annotation method. And SelfMix is applied on intra-sentence triplet extraction and cross-sentence triplet extraction stages. In intra-sentence triplet extraction stage, the loss values are used to train a binary hybrid Gaussian model that distinguishes correct classified data from misclassified data. Correctly classified and possibly misclassified triples are generated after this stage. For “possibly misclassified” triples, the original label is no longer used. But the predicted result of the model is used as a soft label to adapt to noise. In cross-sentence triplet extraction stage, SelfMix is applied for predicting, classifying, and annotating all sentence pairs, which results in insufficient GPU resources in the system. This process is similar to the processing of intra-sentence triplet extraction, but soft labels instead of wrong labels are used here. The labels that are related to the sentence pairs during subsequent training are inputs in the model.

### 3.3 Joint extraction of entity and relation model

**Figure 3.**
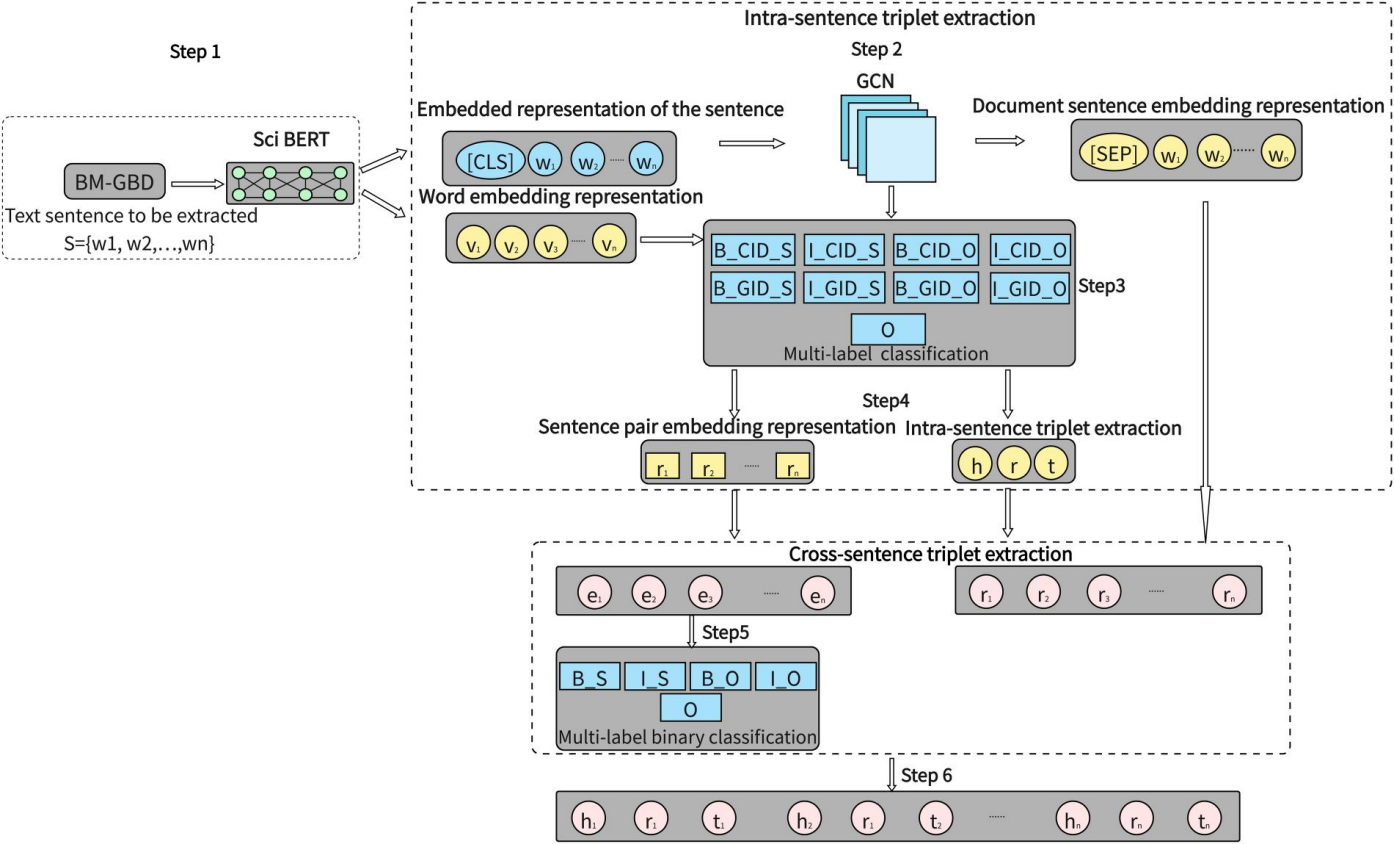
Architecture process of joint extraction model.

The joint extraction of entity and relation model is divided into six steps, which are shown clearly in Fig. 3. Step1: BM-GBD dataset is input into Sci Fine-tuning BERT (Sci BERT) to get two kinds of embedding representations: sentence embedding representations and word vector representations. Step2: The sentence embedding representations are input into the GCN (Yao *et al.* 2019) to obtain the sentence pair embedding representations. And the document sentence embedding representations are obtained. Step3: Joint extraction of entity and relation based on a novel tagging scheme (Zheng *et al.* 2017), complete the intra-sentence triplet extraction. GCN conducts multilabel classification of the sentence pair embedded representations in obtained sentences to determine whether the sentences contain cross-sentence triples. The “BIO” tag principle is adopted in this step, and multiple entities can be resolved based on tag resolution. The “BIO” label table is shown in Table 2. Step4: The relation between sentence pairs is extracted on the sentence pair embedding representations. Step5: “BIO” label principle is used for multilabel classification of entities cross-sentence triplet extraction. A new “BIO” label table is shown in Table 3. Step6: The binary group extraction task for entities and relations achieves the extraction of cross-sentence triples, which is based on the characteristics and classification results in Step4 and Step5.

**Table 2.** “BIO” label table in joint extraction model.

Label	Description
B_CID_S	Head entity start label for chemical disease relationships
I_CID_S	Head entity middle label for chemical disease relationships
B_CID_O	Tail entity start label for chemical disease relationships
I_CID_O	Tail entity middle label for chemical disease relationships
B_GID_S	Head entity start label for genetic disease relationships
I_GID_S	Head entity middle label for genetic disease relationships
B_GID_O	Tail entity start label for genetic disease relationships
I_GID_O	Tail entity middle label for genetic disease relationships
O	No labels

**Table 3.** A new “BIO” label table in joint extraction model.

Label	Description
B_S	The start label of the head entity
I_S	The middle label of the head entity
B_O	The start label of the tail entity
I_O	The middle label of the tail entity
O	No labels

#### 3.3.1 Sentence embedding representations

GCN is applied to output two embedding representations: document sentence embedding representations and sentence pairs embedding representations. GCN considers the sentence as a node in the graph. The input in this graph is only node *V= [v_1_, ····, v_n_]*, without edges, where each *V_i_* is a d-dimensional vector, representing the feature embedding representation of the node *i*. The edge of the input graph is automatically constructed by GCN to process the borderless graph structure. This approach supports the model to effectively learn relations between sentences without explicit edge information.

In the graph learning module of PICK (Yu *et al.* 2021), a semi-supervised learning method based on GCN is proposed. For any two nodes *vi, vj*, the higher is the similarity between the two nodes, the greater is the weight *e_ij_*. Equations (3.1) and (3.2) give reasonable edge weights in the graph and facilitate the subsequent calculation process of graph convolution, where $\sum_{j=1}^{n}E_{ij}=1,E_{ij}>0$.

(3.1) $$e_{ij}=\mathrm{LeakRelu}(w_{i}^{T}|v_{i}-v_{j}|)$$

(3.2) $$E_{ij}=\mathrm{softmax}(e_{ij})$$

The formula for calculating the loss value based on the network structure is shown in (3.3):

(3.3) $$L_{\mathrm{GL}}=\frac{1}{n^{2}}\sum_{i,j}^{n}\;e^{\left(E_{ij}+\eta {∥{∥v}_{i}∥-v_{j}∥}_{2}^{2}\right)}+\gamma ∥E{∥}_{F}^{2}$$

After learning the structure of the graph network, the model constructs the edge of the input graph by itself. Then, the input *G = {V*, *E}* in the GCN is carried out. The embedding representation of each node is shown in (3.4) and (3.5):

(3.4) $$h_{ij}^{l}=\sigma \left(W_{v_{i}h}^{l}v_{i}^{l}+W_{v_{j}h}^{l}v_{j}^{l}+b^{l}\right)$$

(3.5) $$v_{i}^{l+1}=\sigma \left(E_{i}h_{i}^{l}W^{l}\right)$$

For the classification task of an edge, the embedded representations of node pairs in the last layer is shown in (3.6), the *α_ij_* is used for subsequent downstream tasks:

(3.6) $$\alpha_{ij}=\sigma \left(W_{\alpha }h_{ij}^{l}\right)$$

The hyperparameters setting in the GCN are as follows: layers* = *2, *γ = *1, and *η = *1.

#### 3.3.2 Multi-label classification

After a series of calculations, multilabel classification algorithm-ATLOP is used to discover whether there is potential cross-sentence relation in a sentence pair. When dealing with complex tasks such as cross-sentence triplet extraction judgment, some data have multiple labels at the same time. The traditional processing method treats each label as an independent binary classification problem, and selects the corresponding threshold value according to the model’s fit to the data. However, choosing a fixed threshold may be unable to accurately reflect the logical relationship between two complex sentences. To overcome this limitation in practical application, Zhou *et al.* (2021) proposed the ATLOP for document level relation extraction, which had two techniques: adaptive thresholding and localized context pooling. The adaptive thresholding technique replaces the global threshold in multilabel classification with a learnable threshold class that decides the best threshold for each entity pair. The localized context pooling utilizes pre-trained attention heads to locate relevant context for entity pairs and thus helps in alleviating the multi-entity problem. ATLOP splits the labels of entity into two subsets: positive classes *P_T_* and negative classes *N_T_*. If an entity pair is classified correctly, the logit_*r*_ of positive classes should be higher than the threshold while those of negative classes should be lower.

In this way, ATLOP divides the calculation of the loss value into two parts: the loss is calculated for the positive sample class, as shown in (3.7), and the loss is calculated for the negative sample class, as shown in (3.8).

(3.7) $$L_{1}=-\sum_{r\in P_{T}}\;l\mathrm{og}\left(\frac{e^{{\mathrm{logit}}_{r}}}{\sum_{r^{\prime }\in P_{T}\cup \{\mathrm{TH}\}}\;e^{\left({\mathrm{logit}}_{r^{\prime }}\right)}}\right)$$

(3.8) $$L_{2}=-\sum \log\left(\frac{e^{{\mathrm{logit}}_{\mathrm{TH}}}}{\sum_{r^{\prime }\in N_{T}\cup \{\mathrm{TH}\}}\;e^{\left({\mathrm{logit}}_{r}\right)}}\right)$$

(3.9) L=αL1+1-αL2 α∈(0,1)

where logit_*r*_ represents the prediction probability of label *i*, *P_T_* represents the “positive sample label” set, *N_T_* represents the “negative sample label” set, and TH represents the threshold label. The final loss value is the weighted sum of the two parts, as shown in (3.9).

In addition, a key parameter in the ATLOP algorithm is *α*, which is used to balance the weight of positive and negative sample class losses. The experiment results show that by adjusting the value of *α*, the tradeoff between the recall rate and the accuracy of the model are effectively carried out. In this study, a balance of positive and negative sample loss is acquired when the value of *α* equals 0.5.

This loss calculation method allows the model to better understand and learn the characteristics of the data during the training process, and the model more accurately determine whether there are potential cross-sentence triples in the sentence pairs.

#### 3.3.3 Cross-sentence triplet extraction

A binary group extraction model consists of two binary group extraction: the chemical disease relations bivariate extraction and the gene disease relations bivariate extraction. More accurate entity relation identification is achieved by classifying the linear layer and softmax layer of the entity relation binary.

The design of the binary extraction has highlights in order to obtain the efficient cross-sentence triplet extraction from the text. First, the model in this paper focuses on fusing multiple different features from the joint extraction when processing the data. This fusion strategy makes full use of the comprehensive information generated in the model, and enhances the model’s ability to understand and capture the cross-sentence triplet extraction structure. Second, the joint extraction of entity relation process does not involve the direct identification of relation types. Because the kinds of relations involved in the specific cross-sentence triples in each sentence pair have been determined from Step1 to Step5, Step6 focuses on precisely extracting the entity relations of these predefined relation types. The feature addition method is adopted in the experiment for feature fusion based on the limitation of hardware conditions. While this approach may not be as effective as feature splicing in terms of information retention, it provides the necessary advantages in terms of reducing model complexity and adapting to hardware conditions, so the approach makes the model trains properly in resource-constrained environments.

In addition, CRF (Yu and Fan 2020) during the model training enhances the accuracy of the model in the label prediction process by introducing the transformation relations between different labels as a prior knowledge. CRF plays a key role as a loss function specialized for single label classification. However, due to the complexity of the deep learning model and the multiple iterations required to achieve better fitting results, the relevant parameters of standard CRF suffers from a loss value explosion. Local regularization CRF(L-CRF) is adopted to avoid the loss value explosion of CRF parameters after multiple iterations while maintaining the sparsity of the model. The calculation formula of L-CRF is shown in (3.10):

(3.10) $$\begin{array}{c} L_{\mathrm{crf}}=\log Z(x)-\left(h(y_{1};x)+\sum_{t=1}^{n-1}\;[g(y_{t},y_{t+1}+h(y_{t+1};x)]\right)\\ +l_{1}\mathrm{norm}(g)+l_{2}\mathrm{norm}(h) \end{array}$$

## 4 Discussion

### 4.1 Dataset

BM_GBD is a self-built dataset based on the BC5CDR and GDA datasets in this study. The BC5CDR dataset focuses on chemical-disease relationships, and the GDA (Gene-Disease Associations) dataset centers around gene-disease relationships. The annotation process of BM_GBD dataset includes two stages to integrate BC5CDR and GDA. In first stage, part of the data is manually annotated by random sampling, and three parts are formed: BM_GBD_CDR (chemical-disease relationships), BM_GBD_GDA (gene-disease relationships), and BM_GBD_N (unannotated data). In second stage, a semi-supervised learning method is employed. First, a chemical-disease relationship model is trained using BM_GBD_CDR, then this model predicts and supplements chemical-disease relationships in BM_GBD_GDA and BM_GBD_N. Similarly, a gene-disease relationship model is trained using BM_GBD_GDA and predicts BM_GBD_CDR and BM_GBD_N to complete gene-disease relationships. Ultimately, a comprehensive BM_GBD dataset is formed through these two stages of annotation, encompassing both chemical-disease and gene-disease triplet relationships. BM_GBD information is shown in Table 4: CID represents chemical-disease relationships and GID represents gene-disease relationships.

**Table 4.** BM_GBD information.

Dataset	Texts number	CID	GID
Train	2396	2713	2326
Validation	615	1263	964
Test	615	1584	1268

The following datasets are also used for model performance evaluation. Sci ERC (Scientific Information Extraction with Robust Methods and Applications) is a dataset for information extraction from scientific literature. New York Times (NYT) is a common dataset for relation extraction between entities from news articles. ACE04(Automatic Content Extraction) and ACE05 are two annotated datasets for entity and relation extraction.

### 4.2 Performance evaluation

In this study, F1-Score (F1), accuracy, precision and recall are the classification indicators to measure the final results. The experiment results based on the Sci BERT are shown compared with BERT in Table 5. The F1, accuracy, precision and recall have all reached 0.96. The results indicate that the stability and generalization ability of Sci BERT are excellent.

**Table 5.** Sci BERT and BERT experiment results comparison.

Model	F1	Accuracy	Precision	Recall
BERT	0.94	0.94	0.94	0.94
Sci BERT	**0.96**	**0.96**	**0.96**	**0.96**

Bold values indicate the best performance for each metric across the models.

In order to verify the validity of the proposed model, the joint extraction performance is compared on multiple public datasets. The experiment results are shown in Table 6. All values in the table represent the F1 of the model in the corresponding dataset. In the comparing experimental results, all values for the self-built BM_GBD dataset are from independent experiments. The experiment comparison results verify that the model in this study shows significant performance improvement on multiple datasets. In BM_GBD, Sci ERC and NYT datasets, our model outperforms all the baselines in both partial matching and exact matching and achieves 4.7%, 0.4%, and 4.7% improvements over localized context pooling method (Zhong and Chen 2020), respectively. The baseline models were limited by the initial stage of deep learning technology and hardware conditions, so the number of parameters was relatively small and there was a lack of effective language models to mine deep semantic features. These models perform poorly when dealing with technical literature that requires knowledge of vertical domains. Especially on datasets, such as ACE04 and ACE05, the fitting ability of the model is obviously interfered with a large amount of labeling noise. Zhong and Chen (2020) introduced localized context pooling as a method to capture context-specific information for individual entity pairs, leading to significant performance improvements compared to existing methods on benchmark datasets. Therefore, the SelfMix is introduced into the model in this study, and the model performance is significantly improved. Compared with the current excellent models, our model designed performs well on the Sci ERC dataset and the self-labeled BM_GBD, which is mainly due to the feature extraction optimization for the long-distance information dependence problem of long text.

**Table 6.** Comparison of F1 values on different models.

Model	BM_GBD	Sci ERC	NYT	ACE04	ACE05
Qi Li and Heng Ji (2014)	23.5^*^	12.6^*^	45.3^*^	45.3	49.5
Miwa and Bansal (2016)	25.4^*^	20.0^*^	43.4^*^	43.2^*^	50.1^*^
Katiyar and Cardie (2017)	15.7^*^	18.5	48.6^*^	45.7	53.6
Zheng *et al.* (2017)	21.1^*^	19.2^*^	52	48.8^*^	57.5
Yu *et al.* (2021)	65.1^*^	33.9^*^	59	60.5^*^	59.1^*^
Zhong and Chen (2020)	70.8^*^	36.8	86.2^*^	**67**	**62.2**
Sci BERT	71.1^*^	33.9^*^	88.3^*^	60.4^*^	58.0^*^
Sci BERT+SelfMix	74.3^*^	34.6^*^	90.6^*^	64.0^*^	61.3^*^
Sci BERT+CRF	73.4^*^	33.8^*^	88.3^*^	62.7^*^	59.2^*^
Sci BERT+L-CRF	73.5^*^	34.5^*^	89.8^*^	62.4^*^	57.0^*^
Sci BERT+SelfMix+L-CRF	75.5^*^ ^∗^	37.2^*^	90.9^*^	66.8^*^	60.2^*^

The results marked with “*” are from our independent experiments, since some researches don’t provide results for related datasets, while those without “*” are directly cited from corresponding papers.

Bold values indicate the best performance for each metric across the models.

We further analyze the effect of proposed components in joint extraction of entity and relation model in this article. We observe that Sci BERT+SelfMix leads to performance gains over Sci BERT. Furthermore, the model Sci BERT+L-CRF outperforms Sci BERT+CRF. Finally, Sci BERT+SelfMix +L-CRF performance even further, suggesting that both components are important in the model of this article.

In general, on the ground of LLM-enhanced KG, the model designed in this paper has reached the optimal or sub-optimal level on all datasets, especially on the joint extraction task of scientific and technological long literature, which shows its potential ability. Through the effective processing of long text and domain-specific knowledge, the model not only improves the extraction performance, but also shows the application prospect of deep learning in complex text processing tasks.

## 5 Conclusion

This study aims to explore how to extract entity and relation information from biomedical science literature automatically and transform these unstructured scientific texts into structured knowledge, which is of great significance for the construction of KG in downstream tasks. Sci BERT model is used to implement named entity recognition and sentence pair prediction, providing a basis for downstream tasks of joint extraction of entities and relationships. The joint extraction of entity and relation model designed in this paper combines based on a novel tagging scheme joint extraction method and GCN, which not only effectively deals with entity and relation in text, but also preliminarily explores potential cross-sentence extraction triples. The SelfMix and L-CRF are adopted in this study to reduce the impact of label noise on model training and ensure the stability and accuracy of the model.

The experiment results show that the model designed in this study not only deeply understands the full text information, but also accurately grasps the logical relation between sentences, demonstrating its excellent ability in complex text processing. Our study successfully achieves accurate extraction of chemical disease triples and genetic disease triples from biomedical science literature, and adopts semi-supervised label to greatly improve the efficiency on joint extraction of entity and relation. At the same time, the results of this study play a key role in the construction of KG for downstream tasks. By accurately extracting triplet information from the scientific and technological literature and structuring this information, we build a rich and accurate knowledge base based on this study. These works not only speed up biomedical researchers’ access to the core content of the literature, but also maintain and build a more comprehensive and accurate KG. Therefore, the results of this study not only promote the development of literature information processing in the field of biomedicine, but also provide a new perspective and method for the application of deep learning in related fields.

## Data Availability

The data underlying this article will be shared on reasonable request to the corresponding author.
